# Supplementary material for: MetaRibo-Seq measures translation in microbiomes
Source: Nat Commun. 2020 Jun 29;11:3268. doi: 10.1038/s41467-020-17081-z (PMC7324362; doi:10.1038/s41467-020-17081-z)
Supplement: Supplementary file 10 — Supplementary Data 7 [file 41467_2020_17081_MOESM10_ESM.zip › File2/Confidence_High_Taxonomy/276608_out.krona.html]

Javascript must be enabled to view this page.

members
magnitude
magnitudeUnassigned
count
unassigned
taxon
rank

276608\_out

68

superkingdom
68
2

phylum
1239
68

class
68
186801

68
186802
order

family
68
186803

841
68
genus


SRS013158\_contig\_number\_4575SRS013940\_contig\_number\_10159SRS013951\_contig\_number\_2045SRS014287\_contig\_number\_18977SRS014412\_contig\_number\_7318SRS014613\_contig\_number\_17087SRS014855\_contig\_number\_12099SRS014923\_contig\_number\_41541SRS015065\_contig\_number\_13892SRS015190\_contig\_number\_12520SRS015264\_contig\_number\_11145SRS015578\_contig\_number\_30236SRS015579\_contig\_number\_24678SRS015890\_contig\_number\_19928SRS015960\_contig\_number\_17670SRS017821\_contig\_number\_15202SRS018984\_contig\_number\_23991SRS019068\_contig\_number\_38222SRS019267\_contig\_number\_6107SRS019397\_contig\_number\_13169SRS024388\_contig\_number\_contig-100\_46.75102SRS024435\_contig\_number\_42688SRS024549\_contig\_number\_contig-100\_221.30416SRS043001\_contig\_number\_8391SRS043411\_contig\_number\_15543SRS043701\_contig\_number\_17678SRS045739\_contig\_number\_7693SRS049900\_contig\_number\_7995SRS050422\_contig\_number\_34941SRS050925\_contig\_number\_17807SRS052027\_contig\_number\_8145SRS053335\_contig\_number\_11398SRS054956\_contig\_number\_16864SRS055017\_contig\_number\_34308SRS056273\_contig\_number\_33467SRS056519\_contig\_number\_18087SRS058070\_contig\_number\_27715SRS063040\_contig\_number\_contig-100\_1724.239524SRS063518\_contig\_number\_6828SRS065504\_contig\_number\_9055SRS075341\_contig\_number\_22119SRS075716\_contig\_number\_1982SRS076756\_contig\_number\_3035SRS098061\_contig\_number\_1141SRS098827\_contig\_number\_11557SRS098881\_contig\_number\_23313SRS101433\_contig\_number\_7219SRS104084\_contig\_number\_4221SRS1041038\_contig\_number\_9918SRS1041130\_contig\_number\_4814SRS104485\_contig\_number\_2469SRS104975\_contig\_number\_8070SRS105153\_contig\_number\_30519SRS1055034\_contig\_number\_11788SRS142503\_contig\_number\_13306SRS142781\_contig\_number\_9145SRS143070\_contig\_number\_16194SRS143598\_contig\_number\_21281SRS143722\_contig\_number\_4842SRS143780\_contig\_number\_28753SRS144362\_contig\_number\_36240SRS144714\_contig\_number\_13791SRS147919\_contig\_number\_18387SRS148091\_contig\_number\_8556SRS148424\_contig\_number\_30169SRS149181\_contig\_number\_27105SRS149879\_contig\_number\_24506SRS893252\_contig\_number\_7379
species
68
166486
